# Registration with Primary Health Care and COVID-19 mortality: cohort of diabetics from five administrative health regions in the city of Rio de Janeiro, Brazil, 2020–2021

**DOI:** 10.1590/1980-549720230039.2

**Published:** 2023-09-18

**Authors:** Jéssica Chagas de Almeida, Natalia Santana Paiva, Gerusa Gibson, Leonardo Soares Bastos, Roberto de Andrade Medronho, Katia Vergetti Bloch

**Affiliations:** I Universidade Federal do Rio de Janeiro, Instituto de Estudos em Saúde Coletiva – Rio de Janeiro (RJ), Brasil.; II Fundação Oswaldo Cruz – Rio de Janeiro (RJ), Brasil.

**Keywords:** COVID-19, SARS-CoV-2, Diabetes mellitus, Noncommunicable diseases, Primary health care, Health surveillance, COVID-19, SARS-CoV-2, Diabetes mellitus, Doenças crônicas não transmissíveis, Atenção primária à saúde, Vigilância em saúde

## Abstract

**Objective:**

The present study carried out an analysis of survival according to the status of registration with Primary Health Care (PHC) and of factors associated with death from COVID-19, in cases residing in Programmatic Area 3.1 (PA3.1) with a diagnosis of diabetes (in the notification form or in the electronic medical record), of the Municipality of Rio de Janeiro (RJ), Brazil, in 2020–2021.

**Methods:**

A probabilistic linkage of databases was performed based on information on cases notified as COVID-19 and data from the electronic medical records of people living with diabetes. A survival analysis was carried out, using the Cox regression model stratified by age group and adjusted for confounding variables.

**Results:**

Individuals registered with the PHC of PA3.1 had almost twice the risk of death from COVID-19 (adjusted hazard ratio [HRadj]=1.91) when compared to those unregistered. This association was stronger in individuals aged 18 to 59 years registered with the PHC (HRadj=2.82) than in individuals aged 60 years or over (HRadj=1.56).

**Conclusion:**

Surveillance strategies for identifying and adequately monitoring higher-risk groups, among individuals living with diabetes, within the scope of Primary Health Care, can contribute to reducing mortality from COVID-19.

## RESUMO

**Objetivo:**

O presente estudo realizou uma análise de sobrevivência segundo situação de cadastro na Atenção Primária à Saúde (APS) e de fatores associados ao óbito por COVID-19, nos casos residentes da Área Programática 3.1 (AP3.1) com diagnóstico de diabetes (na ficha de notificação ou no prontuário eletrônico) do município do Rio de Janeiro, em 2020–2021.

**Métodos:**

Foi realizado relacionamento probabilístico de bases de dados com base nas informações dos casos notificados por COVID-19 e dos dados de prontuário eletrônico de pessoas que vivem com diabetes. Conduziu-se uma análise de sobrevivência, utilizando-se o modelo de regressão de Cox estratificado por faixa etária e ajustando-se por variáveis confundidoras.

**Resultados:**

Verificou-se que indivíduos cadastrados na APS da AP3.1 possuíam risco quase duas vezes maior de óbito por COVID-19 (hazard ratio ajustada — HRaj=1,91) quando comparados aos não cadastrados na APS da AP3.1. Essa associação foi mais forte naqueles com 18 a 59 anos, cadastrados na APS (HRaj=2,82), do que nos de 60 anos ou mais (HRaj=1,56).

**Conclusão:**

Estratégias de vigilância para a identificação e acompanhamento adequado de grupos de maior risco de mortalidade, dentre indivíduos que vivem com DM, no âmbito da APS podem contribuir para a redução da mortalidade em decorrência da COVID-19.

## INTRODUCTION

The Coronavirus Infectious Disease 2019 (COVID-19) is transmitted through direct contact, rapidly spreads, and has considerable potential to lead to death. As of 2020, the SARS-CoV-2 virus has been responsible for the increase in cases of flu-like syndrome (FS) due to COVID-19 and Severe Acute Respiratory Syndrome (SARS) due to COVID-19. Considered a global public health emergency until May 5, 2023, this disease may present mild to moderate clinical manifestations (FS due to COVID-19), which can be handled within the scope of Primary Health Care (PHC), up to severe and critical cases (SARS due to COVID-19), which will probably require hospitalization and ventilatory support^1,2^. Comorbidities, such as cardiovascular diseases, diabetes mellitus (DM), respiratory diseases, among others, contribute as risk factors for death from COVID-19^1,3,4–6^.

From the onset of the COVID-19 pandemic to epidemiological week (EW) 17 of 2023, six million deaths from COVID-19 have been reported worldwide. Brazil ranks fifth with 700 thousand deaths from COVID-19. The municipality of Rio de Janeiro (MRJ), state of Rio de Janeiro, Brazil, until EW 17 of 2023, reported 1,327,889 cases of COVID-19, of which 38,225 were cases of death from the disease^7,8^.

Based on the hypothesis that individuals living with DM, registered with PHC, may have a lower risk of death from COVID-19 when compared with unregistered individuals, the objective of the present study was to perform an analysis of survival and factors associated with death from COVID-19 according to the status of registration with PHC, in cases residing in Programmatic Area 3.1 (PA3.1) notified by COVID-19 with a diagnosis of diabetes (in the notification form or in the electronic medical record), of MRJ.

## METHODS

### Study design and population

This is a retrospective cohort of incident cases of COVID-19 in residents of PA3.1 of MRJ aged 18 years or older and living with diabetes, with date of onset of symptoms in the period between March 1, 2020 and March 31, 2021.

### Inclusion and exclusion criteria

The inclusion criteria were: date of onset of symptoms as of March 1, 2020; individuals aged 18 years or older; residents of PA3.1; and living with DM.

The exclusion criteria were: unavailable date of onset of symptoms; unavailable date of death; categories of variables whose frequencies were less than 5% (for example: Asian/Indigenous ethnicity).

### Study location

The study was conducted in PA3.1, which comprises one of the ten PA of the MRJ. PA3.1 comprises a territory of approximately 86 km^2^, with an estimated population of 871,024 inhabitants (inhab.)^9^. In PA3.1, approximately 40% of the population lives in favela areas. Its territory is home to some of the largest favela complexes in the MRJ: Complexo da Maré (64,094 inhab.), Complexo do Alemão (63,484 inhab.), Complexo da Penha/Vila Cruzeiro (36,862 inhab.), and Vigário Geral/Parada de Lucas (20,570 inhab.)^10^. The territory has six administrative health regions (AHR) and 28 neighborhoods, with 42 health facilities, namely: 14 municipal health centers, 18 family clinics, two polyclinics, and eight hospitals^10^, to serve a population that mostly resides in vulnerable areas.

### Variables and data sources

Notification data on COVID-19 cases recorded between March 2020 and March 2021, entered into the e-SUS Notifica system (a platform used by the Brazilian Unified Health System for notifying and monitoring suspected and confirmed cases of COVID-19) and/or the System for Information and Epidemiological Surveillance of Influenza (*Sistema de Informação e Vigilância Epidemiológica da Gripe –* SIVEP-Gripe), were requested from the State Health Department of Rio de Janeiro. Data on users registered with the electronic medical records of patients until May 2018 in PHC with a diagnosis of DM in PHC units of PA3.1 were requested from the Municipal Health Department of Rio de Janeiro.

The variable time (in days), considered as the outcome, was defined as the period between the date of onset of COVID-19 symptoms and the evolution of the case (death from COVID-19, cure/in treatment). For the survival analysis, death was defined as the event; and “no death” (cure/in treatment), as censoring.

PHC registration was considered as exposure, and the following covariates were incorporated into the regression model to control for confounding: sex — women, men; ethnicity/skin color — white, black (black + mixed race); registered with PHC — no, yes; comorbidities (cardiovascular disease, respiratory disease, kidney disease); and symptoms (fever, dyspnea) — no, yes.

### Linkage of databases

To identify the individuals registered with PHC of PA3.1 who live with DM, the probabilistic record linkage (RL) was performed between the database of the electronic medical records of people living with DM in the PHC of PA3.1 and the database of cases reported by COVID-19 who lived with DM, throughout the state of Rio de Janeiro, between March 1, 2020 and March 31, 2021.

First, the databases went through the standardization stage, with the withdrawal of erroneous records and characters (accents, cedillas, etc.) present in the main variables, such as “name” and “date of birth,” that could interfere in the process of record linkage. Subsequently, they went through the blocking stage, which divides the bases into logical and comparable blocks in order to optimize RL.

It is at this stage that the Soundex of the patient’s and/or mother’s name is performed, creating comparison blocks with the variable date of birth, the patient’s first name and/or the mother’s first name. Record linkage is based on the construction of scores for the different possible pairs and pairing of the records^11^. This is carried out by using the RL^12^, SoundexBR^13^, and other auxiliary packages of the R free software.

### Statistical analysis

Sociodemographic and clinical data of COVID-19 cases were analyzed, stratified by age group (from 18 to 59 years and 60 years or over), to characterize the study population by the distribution of frequencies according to PHC registration. Differences in the proportions of each group were compared using the χ^2^ test. The adopted significance level was 5%.

Subsequently, the survival analysis was performed using the Kaplan-Meier^14^ product-limit estimator in the calculation of the survival curves for exposure stratified by age group, and the log-rank test for comparing them, seeking to test the null hypothesis that the risk of death from COVID-19 is the same for all groups and that these risks are proportional. A 5% significance level was considered.

The crude and adjusted hazard ratio (HR), together with their 95% confidence intervals (95%CI), for all individuals and by age group, were estimated using the simple and multiple Cox regression model, respectively. The statistically significant variables of the simplest regression model, at the 20% level, were included in the multiple model. Characteristics that altered HR by at least 10% were considered confounders. The effect modification was evaluated by the statistical significance of the interaction term included in the model. The adjustment of the model (deviance analysis) was carried out by the likelihood ratio of the proposed model in relation to the saturated model.

The analyses were performed using the R Development Team software^15^ version 4.0.3.

### Ethical aspects

The study was approved (Certificate of Presentation for Ethical Consideration — CAAE 39769220.6.0000.5286) by the Research Ethics Committee (*Comitê de Ética e Pesquisa* – CEP) of the Institute of Studies on Collective Health of Universidade Federal do Rio de Janeiro (IESC/UFRJ) and by the CEP of the Municipal Department of Rio de Janeiro (*Secretaria Municipal do Rio de Janeiro* – SMS/RJ) (CAAE 39769220.6.3001.5279). In addition, it received the consent of the State Department of Health of Rio de Janeiro (*Secretaria Estadual de Saúde do Rio de Janeiro* – SES/RJ) (process SEI-080001/005459/2021) for data dispensation.

## RESULTS

**Figure 1.**
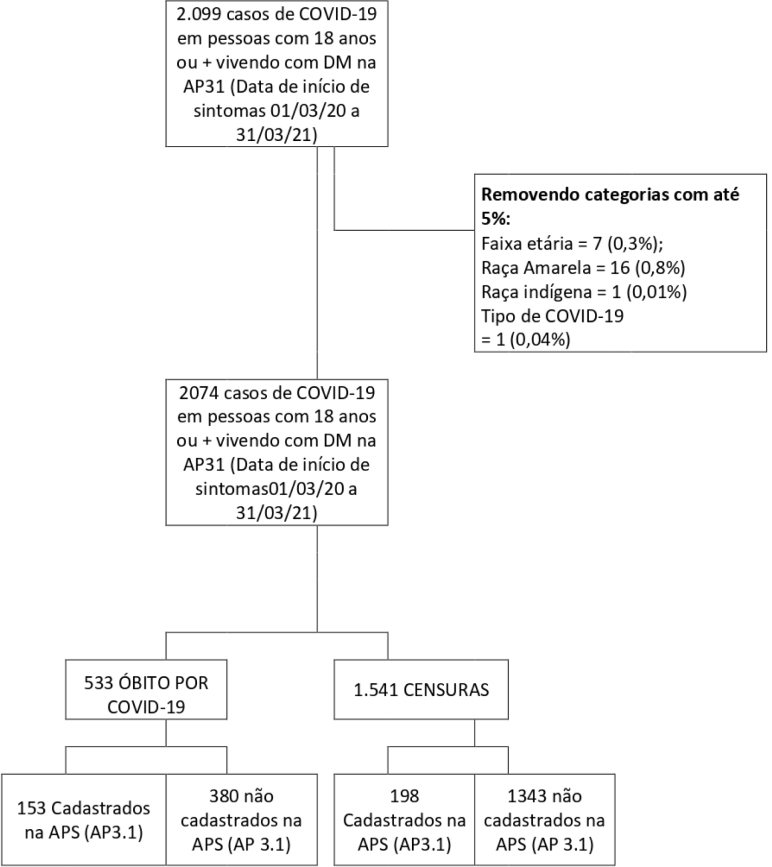
Survival diagram.

Regarding the RL process, at the end of the third stage (record linkage), we identified 369 true pairs (links) and 5,831 possible true pairs (possibility of link). After manual verification, 143 cases were classified as true pairs. By performing the “merge” of the databases, we obtained 512 cases of COVID-19 registered with PHC (as of May 2018) from the total of 2,777 individuals living with DM, residents of PA3.1, notified for COVID-19. The structuring of the database, after the RL, applying the exclusion criteria of the study, resulted in the sample of 2,074 cases of COVID-19 in people living with DM in PA3.1, with 533 deaths from COVID-19 (event) and 1,541 censoring (cure or in treatment). We detail these steps in the diagram of Figure 1.

In Table 1 we present information on COVID-19 cases living with DM, stratified by age group (18 to 59 years and 60 years or over). We observed that 43.6% of those registered died from COVID-19. Women were predominant in both age groups, regardless of their registration status. In the age group of 60 years or over, we identified the highest number of registered people and the highest percentage of deaths from COVID-19 compared with the age group of 18 to 59 years.

**Table 1.** Sociodemographic and clinical characteristics of COVID-19 in people living with diabetes mellitus, residents of Programmatic Area 3.1, confirmed for COVID-19 according to Primary Health Care and age group, March 2020 to March 2021, municipality of Rio de Janeiro (RJ), Brazil.

Characteristics	Overall				18 to 59 years				60 years or over			
	n (%)	PHC Registration		p	n (%)	PHC Registration		p^*^	n (%)	PHC Registration		p^*^
		No (%)	Yes (%)			No (%)	Yes (%)			No (%)	Yes (%)	
Total	2,074 (100.0)	1,723 (83.0)	351 (16.9)		788 (100.0)	702 (89.0)	86 (10.9)		1,286 (100.0)	1,021 (79.4)	265 (20.6)	
Status												
Censoring	1,541 (74.3)	1,343 (77.9)	198 (56.4)	<0.001	661 (83.9)	608 (86.6)	53 (61.6)	<0.001	880 (68.4)	735 (72.0)	145 (54.7)	<0.001
Event	533 (25.7)	380 (22.1)	153 (43.6)		127 (16.1)	94 (13.4)	33 (38.4)		406 (31.6)	286 (28.0)	120 (45.3)	
Type of COVID-19												
FS	875 (42.2)	804 (46.7)	71 (20.2)	<0.001	446 (56.6)	422 (60.1)	24 (27.9)	<0.001	429 (33.4)	382 (37.4)	47 (17.7)	<0.001
SARS	1,199 (57.8)	919 (53.3)	280 (79.8)		342 (43.4)	280 (39.9)	62 (72.1)		857 (66.6)	639 (62.6)	218 (82.3)	
AHR												
AHR10	758 (36.5)	597 (34.6)	161 (45.9)	<0.001	302 (38.3)	262 (37.3)	40 (46.5)	<0.001	456 (35.5)	335 (32.8)	121 (45.7)	<0.001
AHR11	627 (30.2)	517 (30.0)	110 (31.3)		238 (30.2)	212 (30.2)	26 (30.2)		389 (30.2)	305 (29.9)	84 (31.7)	
AHR20	548 (26.4)	502 (29.1)	46 (13.1)		166 (21.1)	159 (22.6)	7 (8.1)		382 (29.7)	343 (33.6)	39 (14.7)	
AHR29	2 (0.1)	0 (0.0)	2 (0.6)		1 (0.1)	0 (0.0)	1 (1.2)		1 (0.1)	0 (0.0)	1 (0.4)	
AHR30	139 (6.7)	107 (6.2)	32 (9.1)		81 (10.3)	69 (9.8)	12 (14.0)		58 (4.5)	38 (3.7)	20 (7.5)	
Sex												
Women	1,137 (54.8)	938 (54.4)	199 (56.7)	0.5	421 (53.4)	375 (53.4)	46 (53.5)	>0.9	716 (55.7)	563 (55.1)	153 (57.7)	0.5
Men	937 (45.2)	785 (45.6)	152 (43.3)		367 (46.6)	327 (46.6)	40 (46.5)		570 (44.3)	458 (44.9)	112 (42.3)	
Age group (in years)												
18 to 39	97 (4.7)	91 (5.3)	6 (1.7)	<0.001	-	-	-		-	-	-	
40 to 49	237 (11.4)	215 (12.5)	22 (6.3)		-	-	-		-	-	-	
50 to 59	454 (21.9)	396 (23.0)	58 (16.5)		-	-	-		-	-	-	
60 or over	1,286 (62.0)	1,021 (59.3)	265 (75.5)		-	-	-		-	-	-	
Ethnicity/skin color												
White	729 (46.3)	585 (47.5)	144 (42.0)	0.081	243 (39.6)	208 (39.2)	35 (42.7)	0.6	486 (50.5)	377 (53.8)	109 (41.8)	0.001
Black	846 (53.7)	647 (52.5)	199 (58.0)		370 (60.4)	323 (60.8)	47 (57.3)		476 (49.5)	324 (46.2)	152 (58.2)	
No information	499	491	8		175	171	4		324	320	4	
No. of comorbidities												
1	890 (42.9)	753 (43.7)	137 (39.0)	0.3	418 (53.0)	378 (53.8)	40 (46.5)	0.4	472 (36.7)	375 (36.7)	97 (36.6)	>0.9
2	1,036 (50.0)	850 (49.3)	186 (53.0)		333 (42.3)	291 (41.5)	42 (48.8)		703 (54.7)	559 (54.8)	144 (54.3)	
3 or more	148 (7.1)	120 (7.0)	28 (8.0)		37 (4.7)	33 (4.7)	4 (4.7)		111 (8.6)	87 (8.5)	24 (9.1)	
Cardiovascular diseases												
No	731 (40.2)	638 (41.7)	93 (32.1)	0.003	372 (52.9)	344 (54.3)	28 (40.0)	0.031	359 (32.2)	294 (32.8)	65 (29.5)	0.4
Yes	1,088 (59.8)	891 (58.3)	197 (67.9)		331 (47.1)	289 (45.7)	42 (60.0)		757 (67.8)	602 (67.2)	155 (70.5)	
No information	255	194	61		85	69	16		170	125	45	
Respiratory diseases												
No	1,962 (94.6)	1,628 (94.5)	334 (95.2)	0.7	753 (95.6)	669 (95.3)	84 (97.7)	0.5	1,209 (94.0)	959 (93.9)	250 (94.3)	>0.9
Yes	112 (5.4)	95 (5.5)	17 (4.8)		35 (4.4)	33 (4.7)	2 (2.3)		77 (6.0)	62 (6.1)	15 (5.7)	
Kidney diseases												
No	1,359 (93.3)	1,177 (94.2)	182 (87.9)	0.001	585 (95.9)	536 (96.2)	49 (92.5)	0.3	774 (91.5)	641 (92.6)	133 (86.4)	0.018
Yes	97 (6.7)	72 (5.8)	25 (12.1)		25 (4.1)	21 (3.8)	4 (7.5)		72 (8.5)	51 (7.4)	21 (13.6)	
No information	618	474	144		178	145	33		440	329	111	
Fever												
No	847 (46.9)	719 (47.2)	128 (45.1)	0.5	367 (50.7)	335 (51.8)	32 (41.6)	0.12	480 (44.3)	384 (43.8)	96 (46.4)	0.6
Yes	960 (53.1)	804 (52.8)	156 (54.9)		357 (49.3)	312 (48.2)	45 (58.4)		603 (55.7)	492 (56.2)	111 (53.6)	
No information	267	200	67		64	55	9		203	145	58	
Dyspnea												
No	817 (43.5)	738 (46.8)	79 (26.0)	<0.001	355 (48.4)	338 (51.2)	17 (23.0)	<0.001	462 (40.3)	400 (43.7)	62 (27.0)	<0.001
Yes	1,063 (56.5)	838 (53.2)	225 (74.0)		379 (51.6)	322 (48.8)	57 (77.0)		684 (59.7)	516 (56.3)	168 (73.0)	
No information	194	147	47		54	42	12		140	105	35	

* p-value; Pearson’s χ^2^ test. PHC: Registration with Primary Health Care until May 2018; FS: flu-like syndrome; SARS: Severe Acute Respiratory Syndrome; AHR: Administrative health region.

We can observe that those registered with PHC developed the most severe form of the disease (SARS due to COVID-19), and the percentage of cases among older people (60 years or over) was higher. Cardiovascular diseases, as well as kidney diseases, were more present in individuals aged 60 years or over, being 70.5% among those registered with PHC. Respiratory disease, ethnicity/skin color, number of comorbidities, and fever were not statistically significant (p<0.05).

**Figure 2.**
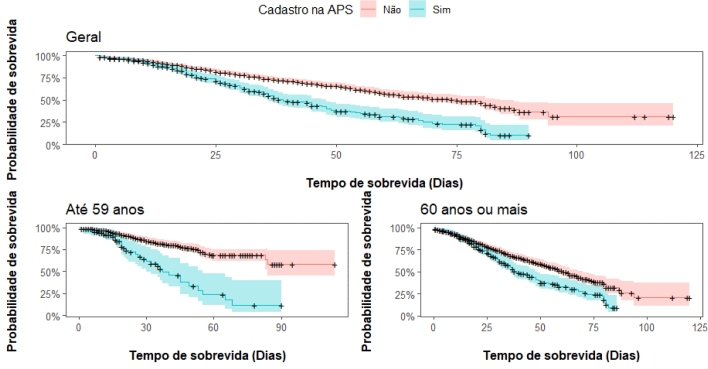
Survival curve, estimated by the Kaplan-Meier method, of COVID-19 cases in people aged 18 years or older living with diabetes mellitus, according to status of registration with Primary Health Care and age group, residents of PA3.1 in the municipality of Rio de Janeiro, from March 2020 to March 2021.

In Figure 2 we present the Kaplan-Meier (KM) curve according to the PHC registration status (see KM curves for sociodemographic variables and risk factors in the Supplementary Material). The survival probability (in days) of COVID-19 cases according to PHC registration, regardless of age group, is higher among unregistered individuals. After analyzing the KM curves according to the PHC registration status by age group, we observed that unregistered individuals aged 60 years or over have a higher survival probability compared to unregistered individuals aged 18 to 59 years. Regarding registered individuals, those aged 60 years or over have a lower survival probability compared to individuals aged 18 to 59 years.

PHC: Primary Health Care

Of the studied covariates, the final multiple model included PHC registration, the outcome of interest and, as adjustment variables, the following covariates: sex, skin color, cardiovascular disease, kidney disease, respiratory disease, fever, and dyspnea (Table 2). In the models (simple and adjusted), it is noteworthy that registered individuals have twice the risk of death from COVID-19 compared to unregistered individuals. As for risk among the age groups, we observed that registered individuals aged 18 to 59 years had a HR of death from COVID-19 in relation to unregistered individuals almost twice as high as the HR of individuals aged 60 years over in the simple model. The HR of death from COVID-19 among registered individuals aged 18 to 59 years remains higher than those aged 60 and over in the adjusted model.

**Table 2.** Crude (HR) and adjusted^*^ (HRaj) hazard ratio and their respective 95% confidence intervals of mortality from COVID-19 in people aged 18 years or older, living with diabetes mellitus, according to Primary Health Care registration status and age group, residents of Programmatic Area 3.1 of the municipality of Rio de Janeiro, March 2020 to March 2021.

	Simple regression model			Multiple regression model^*^		
	Overall	18 to 59 years	60 years or over	Overall	18 to 59 years	60 years or over
	HR (95%CI)	HR (95%CI)	HR (95%CI)	HR (95%CI)	HR (95%CI)	HR (95%CI)
Registration with Primary Health Care						
No	1	1	1	1	1	1
Yes	2.00 (1.6-2.4)	3.36 (2.25-5.00)	1.56 (1.26-1.93)	1.91 (1.38-2.64)	2.82 (1.40-5.67)	1.56 (1.08-2.25)

* Controlled for the covariates: patient’s sex, skin color, cardiovascular disease, kidney disease, respiratory disease, fever, dyspnea (missing data were not included in the Cox analysis). HR: hazard ratio.

## DISCUSSION

From March 2020 to March 2021, 533 deaths from COVID-19 were reported among people living with DM in PA3.1. According to the results, individuals registered with PHC in PA3.1 have approximately twice the risk of death from COVID-19 when compared to unregistered individuals. One of the challenges during the first year of the COVID-19 pandemic in MRJ^16–18^ was the political context, which contributed to the underutilization of PHC and the low level of testing for suspected cases, especially in the most vulnerable areas.

The territory of PA3.1 is composed of four of the main favela complexes of the MRJ, which justifies the importance of health monitoring (at least one medical appointment and one nursing appointment in the period of one year)^19,20^ of the individuals living with DM registered with the PHC, seeking to avoid an imbalance and exacerbation of DM^21^.

When infected by SARS-CoV-2, individuals living with DM can quickly progress to unfavorable clinical conditions, which reduces the survival time^1,22–24^. The registered individuals presented lower survival when compared to unregistered ones. Individuals unregistered with PHC may have more access to private health services, especially during the pandemic.

In the present study, ethnicity/skin color presented a borderline statistical significance. However, ethnicity has been a risk factor for severe cases of COVID-19^22,25^. Arising from discriminatory urbanization, a significant portion of the favela residents are black, without adequate health care for preexisting diseases (such as diabetes or hypertension), with low level of education, without economic support from the government to have a healthy diet, with difficulty in accessing high-quality medical supplies and tests^26^. People living in vulnerable areas have high risks of developing chronic diseases and presenting misinformation about their health status^2,4^. We observed a median time of 20 days from admission to death and, after 65 days of hospitalization, the survival probability was 50%.

Cardiovascular diseases (CVD) can be one of the complications of diabetes and a risk factor for developing severe and critical cases of COVID-19^6^. In this study, approximately 80% of registered individuals evolved to SARS due to COVID-19, and 68% of those registered lived with some CVD, which increases the risk of death, especially when there is no comprehensive follow-up^7,8^ of the health status of the registered user.

When stratifying the model by age group, we observed that individuals aged 18 to 59 years had twice the risk of death from COVID-19^27,28^ when compared with individuals aged 60 years or over. We observed that differences between unregistered and registered individuals regarding the occurrence of risk and prognostic factors is greater in the age group of 18 to 59 years compared with that of 60 years or over (Table 1, making the HR of younger people higher in comparison with the older individuals. This may be due to the profile of the population residing in and registered with the PHC units of PA3.1^29^.

The lack of information from those registered with the PHC of PA3.1, until March 2021, constitutes a limitation of the present study. Due to the change in electronic medical records, throughout the MRJ, in 2018, some variables lost the continuity of collection, resulting in the lack of important information. Regarding the linkage processing, it was necessary to establish a selection for all people notified by COVID-19 in the state of Rio de Janeiro, with DM as a comorbidity. That is, if at the time of filling out the notification form the user’s chronic condition was not informed, the RL was unable to capture this case.

We presented relevant information for the coordination of noncommunicable diseases of MRJ and PA3.1. We expect strategies to be developed to strengthen the DM care line, in addition to working together with the Epidemiological Surveillance, seeking to propose integrated actions aimed at mitigating the occurrence of unfavorable outcomes.

We consider the timely identification of cases and the possible decision-making that culminates in adequate follow-up to be essential^20,30^, accounting PHC responsible for promoting the integrality of care throughout the healthcare network. Hence, the following aspects could be avoided: the exacerbation of the person living with diabetes, the imbalance of glycemic levels, the avoidance of periodic consultations, and the emergence of other factors that may lead to death in an eventual SARS-CoV-2 infection.
